# Professional and governmental policy on community pharmacy: A 10-year policy review and comparative analysis (2008–2017)

**DOI:** 10.1016/j.rcsop.2023.100298

**Published:** 2023-06-28

**Authors:** Evgenia Paloumpi, Piotr Ozieranski, Margaret C. Watson, Matthew D. Jones

**Affiliations:** a Department of Life Sciences, University of Bath, Bath, UK; b Department of Social & Policy Sciences, University of Bath, Bath, UK; c Strathclyde Institute of Pharmacy and Biomedical Sciences, University of Strathclyde, Glasgow, UK

**Keywords:** Community pharmacy services, England, Health policy, Health policy framework

## Abstract

**Background:**

An increased role for community pharmacy might bring considerable value to healthcare systems, for example by relieving workload elsewhere in primary care through the provision of medicines-related services. This requires support from appropriate policy.

**Objective(s):**

To explore the representation of community pharmacy in governmental and professional health policies in England (2008–2017) using the Walt and Gilson policy framework.

**Methods:**

Relevant policies were identified using a systematic search. The content of these policies was analysed using thematic analysis. The transparency of evidence use during the policymaking process was scored in four keys areas using a recognised tool: diagnosis; proposal; implementation; testing and evaluation. Key actors involved in the development of each policy were summarised.

**Results:**

18 governmental policies and 7 pharmacy profession policies were included. Convergence between governmental and professional policy content was identified in 6 areas: healthcare workforce; behaviour and collaborations; utilising technology; urgent care; long-term health conditions; service provision. Divergence was identified in 5 areas: enquiry-driven culture; quality in healthcare; cancer care; mental health care; commissioning. Professional policies were less transparent in their use of evidence and had less documentation of the involvement of key actors, such as professionals and the public.

**Conclusions:**

The profession has limited influence and/or representation in governmental policies. This may be because professional policies did not reflect concerns expressed in governmental policies and had low credibility due to limited stakeholder involvement and transparency about evidence use.

## Highlights

• Governmental and professional policies recognised under-utilisation of community pharmacy.
• They supported community pharmacy's role in long-term and urgent conditions care.
• Professional policies had limited influence and/or representation in governmental policies.
• Professional policies did not reflect governmental concerns about mental health and cancer.
• Professional policies had limited stakeholder involvement and transparency.

## 1 Background

Most healthcare services in England are provided through the tax-funded National Health Service (NHS) model. Community pharmacy is one of 4 fundamental sectors of NHS primary care provided by private businesses under contract to NHS England (a national leadership organisation) alongside general practice, dentistry and eye health. Community pharmacies supply medicines and provide medicines-related and public health services without the need for an appointment. Many have extended opening hours, including at times when general practitioner (GP) services are not available.^1^

Community pharmacies can be found in various locations including high streets, supermarkets, shopping centres, health centres, rural and deprived areas.^2^ In 2020–2021, there were 11,636 community pharmacies in England, of which 60% belonged to multiple contractors (who own 6 or more pharmacies).^3^ It is estimated that about 1.6 million people in England visit a community pharmacy every day.^2^ On average, a person living in England visits a community pharmacy 14 times per year, with 11 of these visits being health-related.^1^ The majority of the population living in England (89.2%) is estimated to have access to a community pharmacy within a 20-minute walk from their home.^4^ However, in the 10% most deprived areas (measured on the Index of Multiple Deprivation), where people might not be able to access general practices, 99.8% of people live within a 20-minute walk of a community pharmacy.^4^

Community pharmacy therefore makes an important contribution towards addressing the health needs of the population in England. However, the context within which it currently operates is complex and challenging. The prevalence of long-term conditions in England is increasing, leading to a large proportion of the population using prescribed medicines.^5^ As a result, the demand for primary healthcare is increasing, leading to profound workload pressures in many services, including community pharmacy and general practice.^5^^,^^6^ However, in December 2015, a 6% reduction of the total funding available for community pharmacy in England was announced, leading to pharmacy representative bodies initiating an ultimately unsuccessful judicial review of the government's decision-making process.^7^ A subsequent economic analysis found that 38% of the community pharmacies were in financial deficit and 52% of owners were planning to sell their businesses.^8^ Subsequently, there was a reduction in the number of community pharmacies in England from 2018 to 2021.^9^

Within this multifactorial environment with many competing challenges, an increased role for community pharmacy has the potential to bring considerable value to healthcare provision, provided that this is supported by appropriate policy.^1^^,^^10^ Previous international policy research has recognised the importance of financial arrangements for the remuneration of cognitive pharmacy services.^11^ Legislative changes and reimbursement mechanisms for non-dispensing community pharmacy services in the UK are thought to constitute an enabling environment for policies aiming to expand the community pharmacists' role.^10^, ^11^, ^12^ However, patient-centred outcomes, quality management, better integration in primary care, and public health have been identified as missing in earlier community pharmacy policy,^10^^,^^12^, ^13^, ^14^ along with a lack of policy-relevant evidence to support the expansion of community pharmacists' role to meet current challenges.^10^

Policies relevant to community pharmacy may be produced by both governmental and pharmacy professional organisations. Compared with each other, these types of organisation are accountable to very different types of stakeholder. They may therefore have different objectives for community pharmacy, so a comparison of their policies and policymaking processes might produce greater understanding of these differences, which could be useful to improve future community pharmacy policy. However, such a comparison has not been previously reported.

## 2 Objectives

The aim of this policy review was to explore the representation of community pharmacy in health policy in England since 2008, using the Walt and Gilson policy framework^15^ to ensure a comprehensive analysis. This framework was designed for health policy analysis and is considered inclusive as it extends beyond policy content.^13^ It suggests that as well as considering the content of policies, research should also investigate their social, economic and political context, the policymaking process, and the actors involved in their development. Therefore, the objectives of this review were to:

1. Compare the content of policies produced by national governmental organisations and the pharmacy profession;
2. Examine the transparency of evidence use during the policymaking process;
3. Identify the actors involved in policymaking.

Analysis of the second objective was carried out using a transparency framework published by 10.13039/100012097Sense About Science and supported by the Institute for Government and Alliance for Useful Evidence, which explores 4 areas of the policymaking process: diagnosis, proposal, implementation, and testing and evaluation.^16^ The changing context for community pharmacy policymaking was also explored, and these findings are presented elsewhere.^17^ The scope of this study was restricted to England (rather than the whole of the United Kingdom) because devolution of responsibility for the NHS to the governments of Scotland, Wales, and Northern Ireland has led to significant policy variation, meaning that governmental and professional policies are only comparable within one country.

## 3 Methods

This study consisted of a systematic search for relevant policies followed by multimethod analysis to address each objective. No ethical approval was required.

### 3.1 Identification of relevant policies

Policies were eligible for inclusion in this study if they met the follow criteria:

• National governmental and professional policies published between April 2008 and July 2017.
• Content specifically related to community pharmacy or considering strategic plans and directions for the future of general healthcare provision in England.

The study period began in 2008 as this was when the influential government policy *Pharmacy in England: building on strengths - delivering the future* was published,^18^ leading to significant change in professional regulation and leadership. The study period then ran until the time when the systematic search for policies was completed.

Initially, websites of national governmental and pharmacy professional organisations were searched. Governmental organisations were defined as national government departments and the national NHS bodies that report to them, with responsibility for community pharmacy. The Department of Health and NHS England were identified as the governmental organisations to be included. There are at least 17 organisations that represent the different areas of the pharmacy profession ranging from professional leadership bodies to trade unions and special interest groups.^19^ The stated purpose of each organisation, as described on their official website, was reviewed to identify three characteristics including whether: members include community pharmacists; the organisation represents community pharmacists; the organisation refers to England geographically (Table S1, supplementary material). Seven organisations fulfilled these criteria, three of which provided publicly available publications from their websites - the Royal Pharmaceutical Society (RPS), Pharmaceutical Services Negotiating Committee (PSNC), and Pharmacy Voice (PV, an organisation that has now disbanded).

Initially, relevant sections (e.g. “publications”, “policies”, or “resources”) of the selected governmental and professional websites were searched and policy documents potentially eligible for inclusion were noted. Free text searching of the websites was also undertaken using the terms “policy”, “community pharmacy” and/or “community pharmacist”. If available, chronological, geographical and status filters were applied.

Additionally, a pharmacy news platform (pharmaceutical-journal.com), the Policy Navigator (navigator.health.org.uk) and the Nuffield Trust policy timeline (nhstimeline.nuffieldtrust.org.uk) were searched in a similar manner.

Search results were initially screened against the eligibility criteria using document titles, before detailed review to select the final included policies. Both steps were completed by one assessor (author EP).

### 3.2 Data extraction

Relevant data from each selected policy were extracted into spreadsheets by one individual (author EP) with independent accuracy checks performed on 3 documents (authors MDJ, PO, MCW). Policy content data extracted verbatim included: aims and objectives, challenges addressed, and recommendations provided. In relation to policymaking, each of the 4 areas of the Sense About Science transparency framework were translated into measurable elements (Table S2, supplementary material). The presence/absence or count of these elements in each policy (and supporting documents such as impact assessments) was extracted. The data extracted regarding policy actors included the presence or absence of specified authors, target audience(s) and professional and public involvement.

### 3.3 Analysis

Data relating to policy content were analysed using thematic analysis based on the 6 stages described by Braun and Clarke.^20^ The aim was to produce a detailed account comparing the aims and objectives, challenges addressed, and recommendations provided by governmental and professional policies. NVivo (version 12; QSR International) was used to facilitate visualisation and theme management. Policy excerpts were read and potential codes noted inductively. Similar codes were then grouped together under a main idea, generating themes and subthemes, with associated definitions. This was discussed and agreed iteratively between team members. Finally, findings and interpretations were recorded in a descriptive account.

Data related to the measurable elements for each of the 4 areas of the Sense About Science transparency framework (Table S2, supplementary material) were scored by a single researcher (author EP) for each policy and 4 documents were cross-checked (author MDJ), according to the following criteria, adapted from those used previously^16^:

• Score 0: Insufficient for level 1.
• Score 1: Some of the elements complete and explained with a degree of transparency.
• Score 2: Most relevant elements complete and/or more transparent.
• Score 3: All relevant elements complete with a more detailed justification and consistency in transparency.

Scores for each of the 4 areas were summarised for governmental or professional policies by the median and interquartile range and compared using Mann-Whitney *U* tests. Policy actors (presence of specific authors, target audience(s), and professional and public involvement) were summarised with descriptive statistics.

## 4 Results

### 4.1 Policies included

The outcome of the policy selection process is summarised in Fig. S1 (supplementary material). A total of 444 records were identified by the initial searches, of which 106 were reviewed in detail after initial screening. After detailed review, 25 policies met the eligibility criteria, 18 from governmental organisations and 7 from the pharmacy profession (Table S3, supplementary material).

### 4.2 Policy content

Three overarching themes were generated by the analysis: ‘improving capacity and capability’, ‘managing chronic conditions’, and ‘providing high quality healthcare services’. The ‘improving capacity and capability’ theme describes the resources required for the optimal performance of the NHS and community pharmacy, covering dimensions such as human, information and intellectual resources, and strategic behaviour and networking. The ‘managing chronic conditions’ theme describes the management of long-term conditions that have an impact on either physical or mental wellbeing, such as cardiovascular diseases, diabetes or cancer. The ‘providing high quality healthcare services’ theme describes the quality of healthcare provided from community pharmacy premises, including dimensions such as care planning, patient-centred care, safety, equity, and services targeted at specific populations. Each theme had sub-themes, which are listed in Table 1. For brevity, these themes are described in detail in a non-peer reviewed report elsewhere,^17^ but areas of similarity and divergence between governmental and professional policies are summarised below in Table 1.

**Table 1:** Summary of themes and sub-themes, and key areas of convergence and divergence between the content of governmental and professional policies.

Theme	Sub-theme	Areas of convergence	Areas of divergence	
			Governmental policies only	Professional policies only
Improving capacity & capability	Healthcare workforce *Present in 15 governmental and 6 professional policies*	Community pharmacists are underutilised	Workload growing faster than staffing	
		Oversupply of pharmacy graduates	Need to increase pharmacy staffing	
		Make more effective use of current workforce, including community pharmacists	Need to maintain staff health & wellbeing	
		Develop pharmacy technicians' role		
		Support community pharmacy training		
		Enhance clinical content of pharmacy undergraduate degree		
	Behaviour & collaborations *Present in 15 governmental and 6 professional policies*	GP workload & recruitment pressures	Variable & growing demand for GP services	Competitive behaviour by GPs
		Need for integrated working between primary care organisations	Stronger relationships with voluntary sector	
		Build stronger relationships with other health professionals & commissioners	Integrated health & social care	
		Pharmacy needs more effective, proactive leadership		
	Utilising technology *Present in 16 governmental and 5 professional policies*	Need for better access to information for community pharmacists, including summary care record	Slow progress implanting digital systems	Cautious that technology might replace pharmacists
		Use technology to support community pharmacy service delivery		
		Use technology to empower patients & support independent living		
	Building an enquiry-driven culture *Present in 15 governmental and 2 professional policies*	Supportive of pharmacy research	Enhance use and collection of evidence	
		Need to record community pharmacy services using metrics	Limited use of pharmacists in clinical research	
			Need stronger evidence-base for community pharmacy interventions	
			Need to focus on supporting research	
	Transforming urgent care *Present in 14 governmental and 5 professional policies*	Increasing pressures in urgent care services	Community pharmacists underutilised in urgent care	
		Need to improve NHS 111 telephone service	Structural reforms needed in urgent care	
		Community pharmacists' inclusion in NHS 111 system	Introduction of NHS 111	
		Enhance community pharmacists' role in urgent care		
Managing chronic conditions	Long-term conditions (LTCs) & medicine use *Present in 12 governmental and 5 professional policies*	Inconsistent care for LTC patients	Community pharmacist roles in specific LTC services	LTCs are the greatest NHS challenge
		High-cost medicines for LTCs	Need for more prevention of LTCs	Community pharmacies to provide LTC services to a specific patient cohort
		Medicines safety challenges in LTCs		
		LTC patients more prone to concurrent mental health conditions		
		Support patients to self-care		
		Pharmacists to implement medicines optimisation		
		Provide integrated care for LTCs		
	Providing care for cancer *Present in 6 governmental and 2 professional policies*	Community pharmacies for access to palliative care medication	Persistent work pressure in cancer services	Multidisciplinary communication needed in palliative care
			Expected rise in cancer prevalence	
			Cancer prevention as a priority	
			Community pharmacy for early detection of cancer	
	Providing care for mental health *Present in 11 governmental and 2 professional policies*	Community pharmacy for promoting healthy living & early detection of mental illness	Need to improve quality of mental health services	
			Expected rise in prevalence of mental illness	
			Need to treat physical & mental health concurrently & equally	
			Specific measures to improve mental health care	
			Services targeted at specific groups	
Providing high quality healthcare services	Service commissioning *Present in 17 governmental and 6 professional policies*	Commissioning of community pharmacy is complex	Community pharmacy not involved in local commissioning	Lack of cohesive approach to community pharmacy commissioning
		Need for a nationally commissioned minor ailments service	Need improved, co-ordinated commissioning of services	Need to alignment community pharmacy and GP contracts
		Commissioning should focus on service quality instead of volume	Nationally commissioning of community pharmacy services, such as smoking cessation	GP-led commissioning could be challenging for community pharmacy
		Community pharmacist involvement in commissioning will enable better integration within health system		National commissioning of community pharmacy influenza vaccination, supervised consumption and emergency hormonal contraception
				Healthy Living Pharmacies as a framework for future commissioning
				National commissioning once services locally evaluated
	Service provision *Present in 16 governmental and 5 professional policies*	The provision of Medicines Use Reviews is problematic & needs redesign	Healthcare services need greater focus on prevention	Community pharmacy services do not fit with national LTC pathways
		Increased pressures on service provision	Services for specific target groups	Inconsistent minor ailments service
		Community pharmacy should support self-care	Greater use of electronic prescription services	Expand immunisation programmes
		Community pharmacy for early detection, prevention and signposting		Host other healthcare professionals in community pharmacies
		Community pharmacy for influenza vaccination		
		Recognition of community pharmacists' role in public health services		
		Need to shift community pharmacy from medicines supply to clinical services		
		Community pharmacy services in patients' homes		
	Quality in healthcare *Present in 15 governmental and 3 professional policies*	Provision or more patient-centred care	Need to improve quality of healthcare services	
			Need to reduce health inequalities	

Substantial similarity between governmental and professional policies (defined as at least half of key concepts being represented in both types of policy, with ‘key concepts’ defined as a small group of codes addressing a related aspect of a theme) was identified in 6 of 11 sub-themes (Table 1). There was joint recognition of the need to make more effective use of the community pharmacy workforce and to support this with improved training, collaboration, and use of technology (sub-themes: healthcare workforce; behaviour and collaborations; utilising technology). Both types of policy also focused on the challenges posed by long-term conditions and urgent care, and how these can be addressed in community pharmacy through improved services and a focus on public health (sub-themes: transforming urgent care; long-term conditions and medicines use; service provision).

Substantial divergence between governmental and professional policies was identified in 5 of 11 sub-themes, with less than half of key concepts represented in both type of policy (Table 1). There was a focus in governmental policies on developing a stronger evidence-based for community pharmacy and making great use of available evidence in developing services that was absent from professional policies (sub-theme: building an enquiry driven culture). Governmental policies described the need to improve the quality of healthcare, such as timely access to services and medication error prevention, whereas professional policies only focused on patient-centred care (sub-theme: quality in healthcare). In relation to cancer care, the governmental policies highlighted work pressures in cancer services, which were anticipated to rise due to increasing prevalence of cancer, and the need for prevention and early detection (sub-theme: providing care for cancer). None of these elements were reflected in any of the professional policies, which focused only on palliative care. In terms of mental health, the governmental policies highlighted 6 future services or areas of need, only one of which was addressed by professional policy (sub-theme: providing care for mental health). In contrast, there was a greater focus on how community pharmacy services are commissioned in professional policies than governmental policies (sub-theme: service commissioning).

### 4.3 Transparency of evidence

The majority of evidence transparency scores for professional policies were 0 or 1, and for governmental policies were 2 or 3 (Table 2 and Figs. S2 and S3, supplementary material). Overall, professional policies were less transparent in their use of evidence in all 4 areas of the policymaking process, and this difference was significant for implementation, and testing and evaluation (Table 2).

**Table 2:** summary of evidence transparency scores for governmental and professional policies.

Evidence transparency area	Median score (interquartile range)		*p*-value⁎
	Governmental policies (*n* = 18)	Professional policies (*n* = 7)	
Diagnosis	2 (1–3)	1 (0–2)	0.08
Proposal	2 (1–3)	1 (1–2)	0.39
Implementation	2 (2–3)	1 (1–3)	0.02
Testing and evaluation	2 (1–2)	0 (0–1)	0.01

⁎ *p*-values were determined using the Mann-Whitney U test.

The lower diagnosis scores for professional policies were partly a consequence of the lack of both a description of the challenges addressed by a policy and a detailed reference list in many professional policies. These elements were more commonly found in governmental policies. Observational studies and previous policies were most often used as evidence for the proposals of both policy types. Publications from the pharmacy profession were used by 5 governmental policies (28%), but every professional policy referred to at least one governmental policy. Less commonly used sources of evidence were randomised-controlled trials, systematic reviews, national statistics, and case studies. Both policy types provided a variety of proposals, but a greater proportion of governmental policies included discussion of costs and benefits. Specific implementation and evaluation plans were more commonly included in governmental policies than professional policies.

### 4.4 Actors

An author was identifiable in 6 governmental (33%) and 2 professional (29%) policies and a target audience was specified in 6 governmental (33%) policies and 5 professional (71%) policies. All but one governmental (94%) and 5 professional (71%) policies provided information about the professionals involved in their development. Finally, public involvement in policymaking was evident in all but 3 governmental policies (83%), but in only 4 professional policies (57%). Overall, all 4 types of actor were identified in 5 governmental (28%) and 3 professional (43%) policies. Table S4 (Supplementary material) summarises the actors identified from each policy.

## 5 Discussion

This review demonstrated similar emphasis between governmental and professional policies for some but not all areas of content. In addition, the policymaking process for professional policies was weaker, with less transparent use of evidence and involvement of professional or public stakeholders. Areas of divergence between governmental and professional policies may be attributable to the differing objectives of these 2 sectors, but also to the less robust policymaking process of the professional sector. Better use of evidence and greater professional and public consultation during professional policymaking might lead to closer alignment of the 2 types of policy. As discussed in the following section, this may lead to faster progress in addressing the many challenges currently facing community pharmacy and ultimately to improved patient care.

### 5.1 Areas of convergence between governmental and professional policies: links with successful implementation

The major areas of convergence included the recognition of mutual challenges such as community pharmacists being underutilised, pressures in urgent care and general practice, and frequent support for community pharmacists' role in long-term conditions management, urgent care provision, use of technology, and further integration in primary care. A shift to community pharmacy services other than dispensing was reflected in policies from both sectors.

Previous studies have also reported policy coverage of these areas. For example, an analysis of the challenges imposed on community pharmacy due to primary care policy reforms in New Zealand found that promoting further integration and collaboration between community pharmacy and primary care had been a common theme in the policy agenda of New Zealand, Australia, United States, Canada, and the UK.^21^ Similarly, New Zealand policies recognised increased demands on services and challenges with GP recruitment.^21^ Studies of policies in England have observed the desire for increased utilisation of community pharmacists, and highlighted the need for a focus on information technology and data sharing, as well as enhancing integration with primary care.^12^^,^^22^

Findings from the current study corroborate other studies that recognise policy in general (as few previous studies have distinguished between policymaking organisations) as a driver for change in community pharmacy services.^23^^,^^24^ The progress achieved in the areas mentioned above illustrates this point. In areas where good alignment was observed between the 2 policy types, further developments have occurred. The mutually recognised role of community pharmacists in providing urgent care may have facilitated the launch in October 2019 of the Community Pharmacist Consultation Service where pharmacists receive referrals from the NHS 111 telephone service.^25^ In addition, community pharmacists have been provided with access to electronic ‘Summary Care Records’ since 2016, following a pilot in 2014.^26^ Community pharmacists are also members of Primary Care Networks, a recently introduced NHS structure for providing collaborative care.^27^

### 5.2 Areas of divergence between governmental and professional policies: contribution to society

Professional policies did not reflect governmental policies regarding the early detection and support for cancer and mental health. The UK health profile for 2019 indicated that cancer is one of the biggest causes of mortality. High levels of depression are also evident, particularly among women.^28^ The RPS has since published policies related to the role of community pharmacy in mental health (2018) and cancer care (2020).^29^^,^^30^

Areas of divergence indicated a partial response to governmental policies from the pharmacy profession not only for the specific diseases described above, but also for broader themes, such as the use and collection of evidence, improving service quality, and tackling health inequalities. An earlier review of the public health agenda of pharmacy policies concluded that the profession focused upon the provision of NHS services without adopting broader public health elements such as social disadvantage and health inequalities.^13^ All these findings of divergence align with previous UK studies describing the need for community pharmacy policy to demonstrate more relevance to society.^13^^,^^31^ The inclusion of all these areas in professional policy in alignment with governmental policy could have resulted in community pharmacy responding to societal need and the political context in a more timely manner. In addition, higher levels of convergence might be achieved through greater responsiveness of governmental policies to proposals from the pharmacy profession, as discussed in the following section.

### 5.3 Policymaking process transparency and actors' involvement

Less than 30% of the governmental policies (*n* = 5) referred to professional policies. The findings of this study cannot fully explain this phenomenon, which is likely to be a result of multiple factors. However, it does suggest that professional policies may not be seen as credible, given their lower scores for evidence transparency (particularly for policy implementation and evaluation), and less frequent documentation of professional and public involvement. Similar variation in the consideration of patient perspectives has been reported for medicines optimisation policies in England.^31^

Planning and implementing a defined agenda for change is a recognised challenge for community pharmacy policy worldwide, while greater commitment to research and evaluation is considered necessary to demonstrate pharmacists' actual rather than potential achievements.^21^ Democratic representation, public deliberation, and expert review are ‘good governance’ in policymaking, adding to the legitimacy and influence of the resultant policies.^32^ Therefore, professional policies might become more credible to governmental organisations with a more transparent and detailed approach to policymaking, especially with regard to implementation, evaluation, and professional and public involvement. A successful example of this was the “Now or Never Report”,^5^ the professional policy that had consistently high scores for transparency and was subsequently referenced by the state in the Community Pharmacy Clinical Services review.^6^ However, it should be noted that professional bodies are smaller and therefore have fewer resources and less policymaking experience than governmental organisations. Similar barriers have been identified to the participation of nurses in health policymaking.^33^ This may explain both the lower transparency scores and omission of key topic such as cancer and mental health in professional policies, and will be a barrier to improving the professional policymaking process in the future. In this context, it is noteworthy that the “Now or Never Report” was published by one of the largest professional organisations, the Royal Pharmaceutical Society.

### 5.4 Strengths and limitations

To the authors' knowledge, this is the first study comparing the content and policymaking process of governmental and professional policies covering the full range of activities of community pharmacists in England. This is also the first application of the Walt and Gilson policy framework to the community pharmacy services field, which ensured a comprehensive analysis of all relevant aspects of policy.

The review is limited to policies relevant to England and published up to 2017. The lack of a comprehensive database of health policies increases the risk that relevant policies were not located and lengthened the research process. Therefore, more recent policies (published after 2017) have been cited in the Discussion where relevant. The analysis could also have been influenced by the research team's professional background, as 3 members are pharmacists. This was addressed by including the non-pharmacist researcher (author PO) at all stages, on-going reference to the original data and the adoption of a reflexive approach to ensure awareness of this potential influence. Finally, due to resource limitations and to increase consistency, policy selection, data extraction, and scoring were performed by only one researcher (author EP), although the wider research team performed a check on a sample of the data extraction and scoring.

### 5.5 Recommendations

Based on these findings, pharmacy professional bodies should work to increase policy credibility by adopting more transparent policymaking processes that respond more quickly to the challenges identified by the state. This should include greater involvement of public and professional stakeholders as equal partners in policymaking and service design. In addition, pharmacy organisations and individual pharmacists should explore the requirements for, and support the development of, community pharmacies' role in providing early detection and support for mental health conditions and cancer, tackling health inequalities, and using and generating evidence. These recommendations will be supported by research comparing the development of governmental and professional policies published after March 2017, or in other countries (especially Scotland, Wales and Northern Ireland) aiming to identify drivers for change and causes of variation.

## 6 Conclusions

Governmental and professional policies recognised the under-utilisation of the community pharmacy sector, supported community pharmacists' role in long-term conditions and urgent care provision, and acknowledged the importance of technology and primary care integration. Professional policies did not reflect the concerns expressed in governmental policies around mental health, cancer, health inequalities and the generation of evidence.

Professional policies were rarely cited in governmental policies. This might be attributed in part to limited transparency and stakeholder involvement in the former. Future professional policies are likely to benefit from addressing these problems and a greater focus on implementation and evaluation.

## Role of the funding source

The funders had no role in the study design, data collection, analysis and interpretation of data; in the writing of the report; and in the decision to submit the article for publication.

## CRediT authorship contribution statement

**Evgenia Paloumpi:** Conceptualization, Methodology, Project administration, Data curation, Formal analysis, Writing – original draft. **Piotr Ozieranski:** Conceptualization, Methodology, Supervision, Writing – review & editing. **Margaret C. Watson:** Conceptualization, Methodology, Supervision, Funding acquisition, Writing – review & editing. **Matthew D. Jones:** Conceptualization, Methodology, Supervision, Funding acquisition, Writing – review & editing.

## Declaration of Competing Interest

None.

## Appendix A Supplementary data

Supplementary material Image 1
