# Supplementary material for: Professional and governmental policy on community pharmacy: A 10-year policy review and comparative analysis (2008–2017)
Source: Explor Res Clin Soc Pharm. 2023 Jun 28;11:100298. doi: 10.1016/j.rcsop.2023.100298 (PMC10339181; doi:10.1016/j.rcsop.2023.100298)
Supplement: Supplementary file 1 — Supplementary material [file mmc1.pdf]

Exploratory Research in Clinical and Social Pharmacy

Community pharmacy professional and governmental policy: a 10-year policy review and comparative analysis (2008-2017)

Supplemental Material

Evgenia Paloumpi[a], Piotr Ozieranski[b], Margaret C. Watson[c] and Matthew D. Jones[d] (corresponding author)

[a]Department of Life Sciences, University of Bath, Bath, UK.

[b]Department of Social & Policy Sciences, University of Bath, Bath, UK.

[c]Strathclyde Institute of Pharmacy and Biomedical Sciences, University of Strathclyde, Glasgow, UK.

[d]Department of Life Sciences, University of Bath, Bath, UK. M.D.Jones@bath.ac.uk, telephone: +44 1225 383829.

Table S1: Pharmacy organisations in the UK and the criteria for their inclusion as policy sources for this review (shaded rows indicate the organisations included).

Organisation	Number of members	Year established	Members include community pharmacists	Represents community pharmacy	Covers England
Pharmaceutical Services Negotiating Committee (PSNC)	11,700 pharmacies	1946	✓	✓	✓
Royal Pharmaceutical Society (RPS)	Over 46,000	1841	✓	✓	✓
Boots Pharmacists' Association (BPA)	1,646	1973	✓	✓	✓
Pharmacy Voice (PV)	Approx. 11,000 pharmacies	2010	✓	✓	✓
National Pharmacy Association (NPA)	Approx. 6,500 pharmacies	1921	✓	✓	✓
Company Chemists' Association (CCA)	Over 6,500 pharmacies	1898	✓	✓	✓
Association of Independent Multiple Pharmacies (AIMp)	132	2002	✓	✓	✓
Guild of Healthcare Pharmacists (GHP)	4,483	1923	✓	x	✓
Academy of Pharmaceutical Sciences (APS)	350	2001	x	x	✓
Pharmacists' Defence Association (PDA)	Over 26,000	2003	✓	x	✓
Primary Care Pharmacy Association (PCPA)	2,470	1999	x	x	✓
Primary and Community Care Pharmacy Network (PCPN)	230	1986	✓	x	✓
Community Pharmacy Wales (CPW)	717 pharmacies	1948	✓	✓	x
Community Pharmacy Scotland (CPS)	1,253 pharmacies	1912	✓	✓	x
Community Pharmacy Northern Ireland (CPNI)	Not disclosed	1948	✓	✓	x
British Pharmaceutical Students' Association (BPSA)	15,000	1942	x	x	✓
Association of British Pharmaceutical Industry (ABPI)	Over 150	1891	x	x	✓

Table S2: Measurable elements examined to score each area of the Sense About Science transparency framework

Diagnosis	Proposal	Implementation	Testing and Evaluation
Presence of clear aims and objectives	Use of systematic reviews as evidence	Presence of specific action plans or tables	Presence of clear plans for measuring success
Presence of clearly defined substantive policy sections	Use of randomised-controlled trials as evidence	Use of consultations with experts or the public	Presence of timetables for evaluation
Number of references used	Use of observational studies as evidence	Presence of plans for further publications	Presence of a separate chapter for evaluation
Number of unavailable documents when the source of reference was searched[1]	Use of national statistics documents as evidence	Presence of specific dates or timeframes for either action plans, consultations or publications	
Number of references that did not direct readers to a specific source, but provided further information as explanations	Use of other policies as evidence	Presence of alternative implementation plans considered	
Presence of citations as footnotes in policy pages	Use of case studies as evidence	Presence of indicators for cost or benefit considerations	
Presence of a separate chapter for challenges	Presence of alternative proposals considered	Presence of a separate chapter for implementation	
	Presence of indicators for cost or benefit considerations		

[1]Unavailable source refers to links that did not work and did not allow identification of the document referenced

Figure S1: Flow diagram of the policy selection process

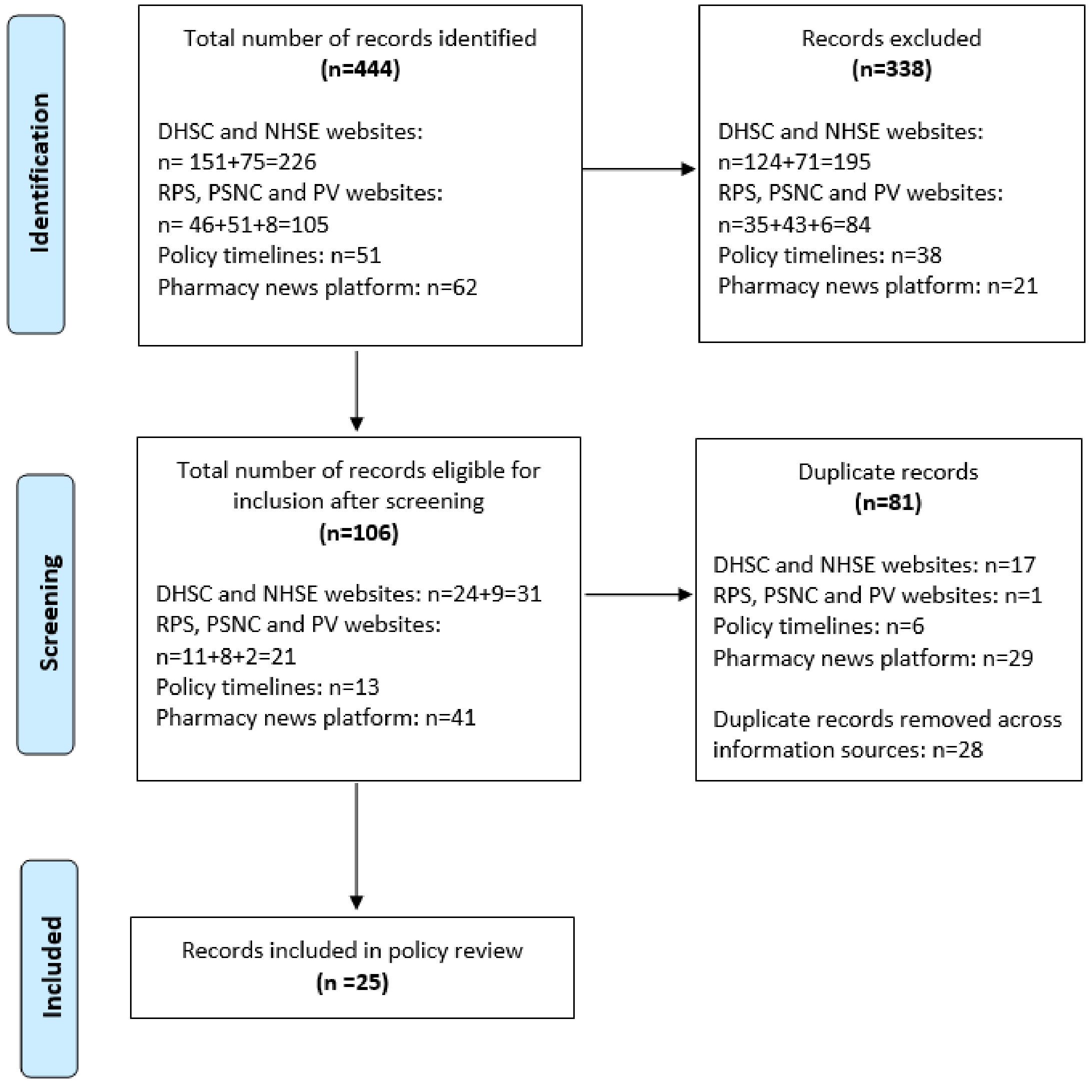

Table S3: Policy documents included in policy review

ID Number	Title	Publication date	Policy origin
S-08-1	Pharmacy in England: Building on strengths - Delivering the future	April 2008	Governmental
S-08-2	High Quality Care for All - NHS next stage review final report	June 2008	Governmental
S-10-3	Equity and Excellence: Liberating the NHS	July 2010	Governmental
S-10-4	Healthy Lives, Healthy People: Our strategy for public health in England	November 2010	Governmental
P-11-5	Community Pharmacy - A blueprint for better health	February 2011	Pharmacy Profession
S-12-6	The Mandate: A mandate from the Government to the NHS Commissioning Board: April 2013 to March 2015	November 2012	Governmental
P-13-7	The vision for NHS Community Pharmacies - The path to improved patient care	August 2013	Pharmacy Profession
S-13-8	A Mandate from Government to NHS England 2014-2015	November 2013	Governmental
S-13-9	High quality care for all, now and for future generations: Transforming urgent and emergency care services in England - Urgent and Emergency Care Review End of Phase 1 Report	November 2013	Governmental
P-13-10	Now or never: Shaping pharmacy for the future	November 2013	Pharmacy Profession
S-14-11	Transforming Primary Care: Safe, proactive, personalised care for those who need it most	April 2014	Governmental
P-14-12	Improving urgent and emergency care through better use of pharmacists	September 2014	Pharmacy Profession
S-14-13	NHS Five Year Forward View (5YFV)	October 2014	Governmental
S-14-14	Community Pharmacy-helping provide better quality and resilient urgent care (Version 2)	November 2014	Governmental
S-14-15	The Government's Mandate to NHS England 2015-16	December 2014	Governmental
S-15-16	Quick Guide: Extending the role of community pharmacy in urgent care	November 2015	Governmental
S-15-17	The Government’s mandate to NHS England for 2016-17	December 2015	Governmental
S-16-18	General Practice Forward View	April 2016	Governmental
P-16-19	Community Pharmacy Forward View	August 2016	Pharmacy Profession
S-16-20	Community pharmacy in 2016-2017 and beyond (Final Package)	October 2016	Governmental
P-16-21	Improving care for people with Long Term Conditions	November 2016	Pharmacy Profession
S-16-22	Community Pharmacy Clinical Services Review	December 2016	Governmental
P-17-23	Community Pharmacy Forward View Part II-Making it happen	January 2017	Pharmacy Profession
S-17-24	The Government's Mandate to NHS England for 2017-18	March 2017	Governmental
S-17-25	Next Steps on the NHS Five Year Forward View	March 2017	Governmental

Figure S2: Evidence transparency scores for governmental policies (n=18)

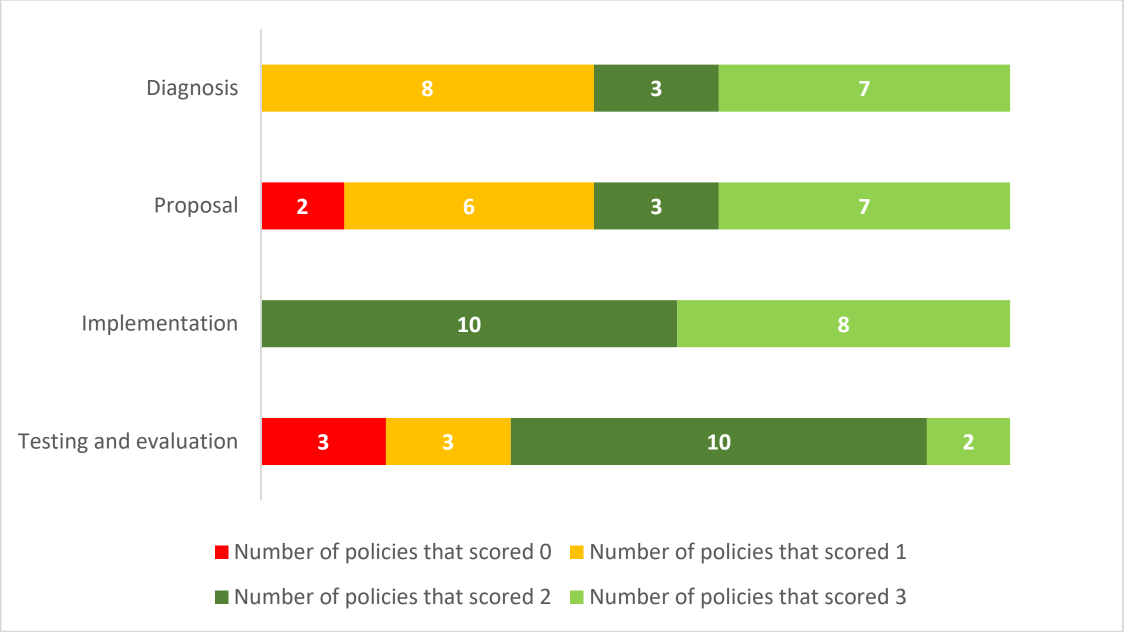

Figure S3: Evidence transparency scores for professional policies (n=7)

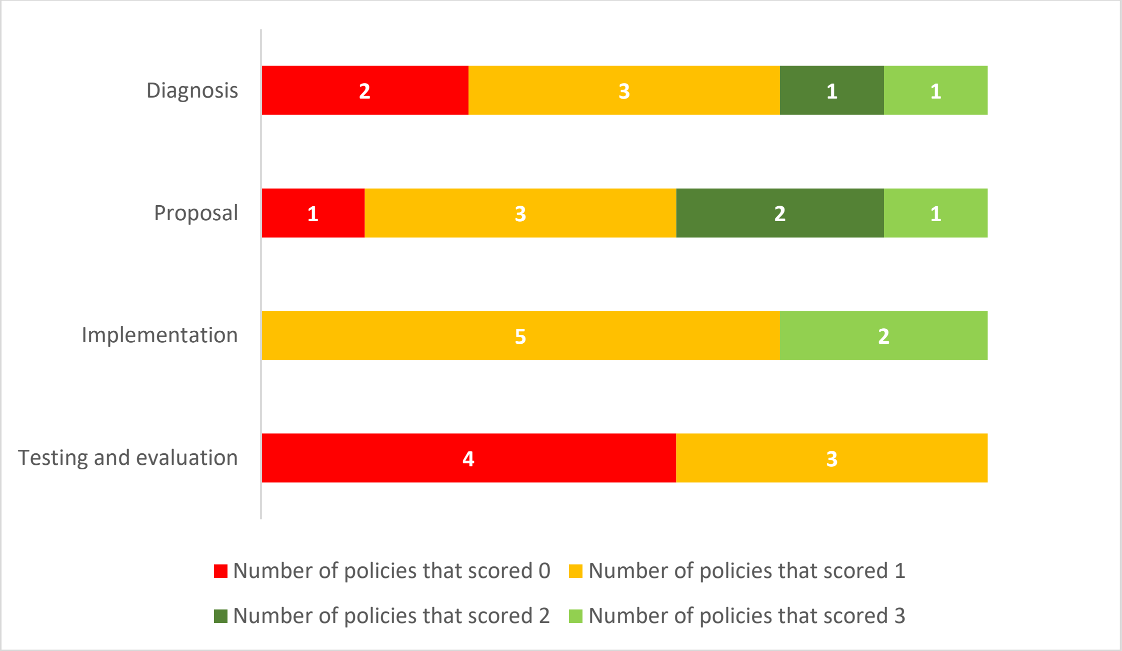

0	Not clearly enough for level 1
1	Some of the relevant fields were complete and explained with a degree of transparency
2	As in level 1 but most relevant fields were complete and/or more transparent
3	As in level 2 but all relevant fields were complete with a more detailed justification and consistency in transparency

Table S4: Actors' identification in each policy

ID number	Policy title	Specified author	Target audience	Professional involvement	Public involvement
S-08-1	Pharmacy in England: building on strengths - delivering the future	✗	✗	✓	✓
S-08-2	High Quality Care for All - NHS next stage review final report	✓	✗	✓	✓
S-10-3	Equity and Excellence: Liberating the NHS	✓	✗	✓	✓
S-10-4	Healthy Lives, Healthy People: Our strategy for public health in England	✗	✗	✓	✓
P-11-5	Community Pharmacy - a blueprint for better health	✗	✗	✓	✓
S-12-6	The Mandate: A mandate from the Government to the NHS Commissioning Board: April 2013 to March 2015	✓	✓	✓	✓
P-13-7	The vision for NHS Community Pharmacies - The path to improved patient care	✓	✓	✓	✗
S-13-8	A Mandate from Government to NHS England 2014-2015	✓	✗	✓	✓
S-13-9	High quality care for all, now and for future generations: Transforming urgent and emergency care services in England - Urgent and Emergency Care Review End of Phase 1 Report	✓	✓	✓	✓
P-13-10	Now or never: shaping pharmacy for the future	✓	✓	✓	✓
S-14-11	Transforming Primary Care: safe, proactive, personalised care for those who need it most	✓	✗	✓	✓
P-14-12	Improving urgent and emergency care through better use of pharmacists	✗	✗	✗	✗
S-14-13	NHS Five Year Forward View (5YFV)	✓	✗	✓	✓
S-14-14	Community Pharmacy-helping provide better quality and resilient urgent care (Version 2)	✓	✓	✓	✓
S-14-15	The Government's Mandate to NHS England 2015-16	✓	✗	✗	✓
S-15-16	Quick Guide: Extending the role of community pharmacy in urgent care	✓	✓	✓	✓
S-15-17	The Government's mandate to NHS England for 2016-17	✗	✗	✓	✓
S-16-18	General Practice Forward View	✓	✗	✓	✗
P-16-19	Community Pharmacy Forward View	✓	✓	✓	✓
S-16-20	Community pharmacy in 2016-2017 and beyond (Final Package)	✓	✓	✓	✓
P-16-21	Improving care for people with Long Term Conditions	✓	✓	✗	✗
S-16-22	Community Pharmacy Clinical Services Review	✓	✓	✓	✗
P-17-23	Community Pharmacy Forward View Part II-Making it happen	✓	✓	✓	✓
S-17-24	The Government's Mandate to NHS England for 2017-18	✓	✗	✓	✗
S-17-25	Next Steps on the NHS Five Year Forward View	✓	✗	✓	✓
